# Cinnamon Essential Oil-Loaded Halloysite Nanotubes Applied in Degradable Film: Characterization and Non-Contact Antimicrobial Activity

**DOI:** 10.3390/polym17091144

**Published:** 2025-04-23

**Authors:** Mingyu Zhou, Yuhang Tian, Shuseng Mo, Can Zhang, Ning Zhuang, Huaming Zheng

**Affiliations:** 1 Hubei Key Lab of Plasma Chemistry and Advanced Materials, Wuhan Institute of Technology, Wuhan 430205, China; 2 Hubei Yihua Degradable New Materials Co., Ltd., Yichang 443005, China

**Keywords:** cinnamon essential oils, halloysite nanotubes, PBAT film, non-contact antimicrobial

## Abstract

To extend food shelf life and reduce plastic pollution, halloysite nanotubes (HNTs) were employed as a carrier to load cinnamon essential oils (CEOs), and the nanotubes were blended with polybutylene adipate co-terephthalate (PBAT) resin to fabricate the film with non-contact antimicrobial activity. The results showed that the HNTs had a high loading efficiency (about 11%) for CEOs. The retention rate of CEOs in HNTs was still 33% after twenty days later, which indicated that the CEOs/HNTs nanoparticles had a long-acting controlled-released effect. The composite films represented excellent mechanical properties and antibacterial effects against *Staphylococcus aureus* and *Escherichia coli* due to the non-contact antimicrobial activity of CEOs. The strawberries remained fresh after five days when the composite film was applied in the packaging of strawberries, which proves that composite films can extend the shelf life of food. Therefore, it has potential application prospects in the food industry.

## 1. Introduction

It is well known that the overuse of traditional plastics for packing materials has caused serious environmental pollution [1]. As people become more aware of the environmental impacts of traditional plastics, there has been a growing trend towards using biobased materials as an alternative to plastic packaging materials, especially degradable bioplastics [2]. Degradable materials mainly include starch-based plastics, polylactic acid (PLA), polybutylene adipate co-terephthalate (PBAT), polybutylene succinate (PBS), and so on. PBAT is one of the best degradable materials in terms of the adherence of degradable bioplastics; it is a copolymer of butylene adipate and butylene terephthalate, commonly referred to as poly(butyleneadipate-co-terephthalate); it can be fully degraded in the condition of natural compost; and does not leave any toxic residues or microplastics [3,4]. Therefore, PBAT films can effectively prevent environmental plastic pollution.

Half of plastic is used in food packaging to prevent decay. Usually, the shelf life of most foods in natural reserves is relatively short, and hence people often use chemical additives to extend their shelf life. However, chemical additives can easily harm health, which motivates researchers to explore alternative methods for prolonging shelf life without compromising health [5]. Essential oils (EOs) can be widely used as natural and safe food preservatives because of their effective antibacterial activity. Zheng [6] utilized carvacrol to inhibit the growth and reproduction of *Escherichia coli* and *Staphylococcus aureus*, resulting in a good effect on the preservation of banana. Kim, J. [7] studied the prolonged insecticidal activity of clove oil-loaded halloysite nanotubes on plodia interpunctella infestation and application in industrial-scale food packaging; and Lee, MH [8] encapsulates thyme oil in allostone nanotubes for antibacterial packaging systems. Due to the broad-spectrum antimicrobial properties and oxidation resistance of cinnamon essential oils (CEOs), there is widespread concern about their ability to maintain fresh food [9]. However, CEOs have limited application in the area of food, mainly because they cannot dissolve in water and often have high volatility. To overcome this defect, methods for using essential oils include preparing liposomes, emulsion, or other loading systems, and using encapsulation carriers to load essential oils to delay their release rate [10,11].

Halloysite nanotubes (HNTs) are a type of natural silicate mineral, primarily composed of silica and alumina, which exhibit excellent biocompatibility [12]. The hollow tubular structure and large aspect ratio of HNTs make them an ideal carrier for slow and controlled release [13]. Asadi [14] exploited the halloysite-loaded inhibitor to improve the protective function of epoxy ester coating, and the results showed that the coating containing halloysite particles had a better protective effect than the pure coating. Meanwhile, halloysite, as a nano-filler, exhibited a strengthening and reinforcing effect. The addition of halloysites into resin could improve the tensile strength and elongation at the break of the material [15].

Therefore, this study selected CEOs as an antibacterial agent and HNTs as a carrier to mix with PBAT matrix resin. Degradable films with a good antimicrobial ability were fabricated using the blown film process. The preservation effect on strawberries was also investigated.

## 2. Materials and Methods

### 2.1. Materials

Polybutylene adipate terephthalate (PBAT, Industrial grade, Zhejiang Huafeng Environmental Protection Materials Co., Ltd., Rui’an, China), halloysite nanotubes (Industrial grade, Shunhe Huatao Factory, Danjiangkou, China), titanate coupling agent (TMC-980, Tianchang Green Chemical Auxiliary Factory, Tianchang, China), stearic acid (SA-1865, Wilmar Oil Technology Co., Ltd., Tianjin, China), and cinnamon essential oils (purity ≥ 90.2%, Shuinan Weiba Medicinal Spice Oil Refinery, Ji’an, China) were used.

### 2.2. Preparation of CEOs/HNTs Nanoparticles

**Purification of HNTs** According to Zheng’s method [6], 10 wt.% HNT suspension was prepared by slowly adding HNT powder into ultrapure water by stirring. Then, 0.05 wt.% sodium hexametaphosphate was added to the suspension, and the mixture was then heated under 60 °C for 30 min. The products were segregated by centrifugation at 4000 rpm for 10 min and washed 3 times by ultrapure water. The purified HNTs were dried at 60 °C for 12 h. Finally, the sample was grinded and filtrated by an 80-mesh sieve.

**Preparation of CEO-loaded HNTs** According to Barman’s method [16], purified HNTs and CEOs with a mass ratio of 1:2 were mixed and ultrasonically dispersed for 30 min. Subsequently, the mixture was transferred into a vacuum jar which was connected to a vacuum pump. One mbar pressure was used to remove air inside the HNTs for 30 min. Finally, the CEOs were allowed to enter the evacuated HNTs under standard atmospheric pressure. The cycle was repeated twice. The CEO/HNT dispersion was segragated by centrifugation at 8000 rpm for 10 min. The precipitate was washed by absolute ethanol to remove CEOs adsorbed on the external surface of the HNTs. Finally, the CEO/HNT nanoparticles were dried overnight at room temperature in an open container.

### 2.3. Processing of CEOs/HNTs/PBAT Biodegradable Films

To enhance the compatibility of halloysite nanotubes (HNTs) with poly(butylene adipate-co-terephthalate) (PBAT) and promote their uniform dispersion within the polymer matrix, a specific amount of titanium coupling agent (4 wt%) and stearic acid (1 wt%) was co-introduced into a high-speed mixer through sequential addition [17,18]. The material temperature was maintained at 70 °C, and the mixture was stirred for 10 min to ensure uniform surface modification. Subsequently, a twin-screw extrusion system (L/D = 48, HK26, Nanjing Keya Chemical Equipment Co., Ltd., Nanjing, China) was employed to prepare the CEOs/HNTs/PBAT biodegradable resin pellets. The mass ratio of CEOs/HNTs to PBAT was set at 1:9. The extrusion process was carried out under a temperature profile of 140 °C, 140 °C, 145 °C, 145 °C, 145 °C, 145 °C, 150 °C, and 150 °C across the screw zones, with a screw speed of 60 rpm. The final film-blowing process was conducted at temperatures of 150 °C, 150 °C, 160 °C, and 160 °C (FB-300, Harbin Hapro Electric Technology Co., Ltd., Harbin, China); the blown film extruder features a die opening diameter of 35 mm and achieves a final bubble diameter of 105 mm during the film formation process.)

### 2.4. Characterization

#### 2.4.1. FT-IR Analysis

The sample was mixed with potassium bromide (KBr) powder at a mass ratio of 1:100, thoroughly ground, and pressed into thin sheets for analysis (Nicolet 6700, Thermo Fisher Scientific Inc., Waltham, MA, USA). Measurements were conducted within the wavenumber range of 500–4000 cm^−1^, with a resolution of 4 cm^−1^ and 64 scans per sample. Pure potassium bromide tablets were prepared and used as the blank control group [19].

#### 2.4.2. The Content of CEOs in Nanoparticles During the Different Storage Periods

The retention rate of cinnamon essential oils (CEOs) in nanoparticles stored at room temperature over different time intervals was measured using a synchronous thermal analyzer (TGA-5500, TA instruments, New Castle, DE, USA). Approximately 5 mg of each sample was weighed and placed into an alumina crucible. The analysis was conducted under a nitrogen atmosphere with a nitrogen flow rate of 40 mL/min. The temperature range was set at 40 °C to 800 °C, with a heating rate of 20 °C/min. The composite films were sealed in disposable sealed bags and stored in a desiccator at room temperature; samples were collected and analyzed on the 0th, 5th, 10th, 15th, and 20th days. In the same way, the loading efficiency of CEOs in PBAT films was also analyzed and determined.

#### 2.4.3. Field Emission Scanning Electron Microscope (FE-SEM)

The CEO-loaded composite film was subjected to brittle fracture in liquid nitrogen. The surface, cross-section, and CEO/HNT nanoparticles were coated with a thin layer of gold via sputter coating. The morphology of the samples was then observed using a Zeiss SIGMA 300 field emission scanning electron microscope (FE-SEM, Oberkochen, Germany) under an accelerating voltage of 3.0 kV.

#### 2.4.4. Transmittance and Barrier Properties of the Films

**Light transmittance performance:** The light transmittance of films was measured using a transmittance/haze tester (WGT-S, Shanghai Jingke Industrial Co., Ltd., Shanghai, China). The surface of each film should be free of wrinkles and contamination. The film was cut into 6 cm × 6 cm pieces, which were fixed onto a fixture for testing. Each group of samples was tested in triplicate, and the data were averaged.

**Water permeability:** The water-permeability coefficient was tested using a water vapor permeability meter (W405L, Guangzhou GBPI Packaging Equipment Co., Ltd., Guangzhou, China). The films were cut into standard pieces of 50.26 cm^2^ and tested under conditions of 38 ± 0.6 °C and a relative humidity of 90 ± 2% RH. Each group of samples was tested three times.

**Oxygen permeability:** The oxygen-permeability coefficient was determinate through coulometry under standard conditions (ASTM D3985-24) using OX-TRAN equipment (2/20 Type, Mocon, Minneapolis, MN, USA), operating with pure oxygen as the permeate gas at 23 °C [20]. The effective permeation area of each sample was 50 cm^2^. The results were adjusted to 1 atm of the partial oxygen pressure gradient.

#### 2.4.5. Mechanical Properties

A universal instrument of mechanical properties (Instron, 5565, Norwood, MA, USA) was used for tensile strength, elongation at break. Films were cut into rectangular shapes with a length of 150 mm and a width of 15 mm, and were then clipped by two clamps on instrument. The thickness of each sample was measured using an electronic thickness gauge, and the average value was obtained after testing five points. The constant extension speed was set at 4 mm/min. The machine direction tensile strength (MD) and transverse direction tensile strength (TD) of each group of samples were tested three times. The tensile strength and elongation at break of the film were calculated using Formulas (1) and (2), respectively.

(1) $$TS=\frac{F}{15\cdot d}$$

where ***TS*** is the tensile strength of the film (MPa), ***F*** is the maximum tensile force during the film stretching process (N), and ***d*** is the thickness of the film (mm).

(2) $$E=\frac{L-L_{0}}{L_{0}}\times 100\%$$

where ***E*** is the elongation at break of the film (%), ***L*** is the length of the film at break (mm), and ***L*_0_** is the initial distance between the two clamps (mm).

#### 2.4.6. Antimicrobial Activity of the Films

The antibacterial effect of the film against *Escherichia coli* and *Staphylococcus aureus* was evaluated using a flatbed counter method. A 0.1 mL aliquot of bacterial suspension with a concentration of 10^5^–10^6^ CFU/mL was added to a test tube containing 9.9 mL of nutrient broth. Then, 0.1 g, 0.15 g, and 0.2 g of composite films were added to separate tubes, while a tube without film served as the control group. Each test tube was placed in a constant-temperature incubator and shaken for 24 h at 37 °C.

Subsequently, a certain volume of the diluted bacterial suspension was spread on an agar plate, which was incubated at 37 °C for 24 h. After incubation, the number of colonies on each petri dish was counted. The bacteriostatic rate was calculated based on the number of colonies according to Formula (3).

(3) $$I=\frac{A_{0}-A_{1}}{A_{0}}$$

where ***A*_0_** is the number of colonies in the control group (without film) and ***A*_1_** is the number of colonies in the presence of the composite films.

#### 2.4.7. Inhibition Zone of the Nanoparticles Experiment

To verify the non-contact antibacterial effect of CEOs/HNTs, we diluted *E. coli* and *S. aureus* to 10^5^ CFU/mL and evenly distributed them on the agar in the culture dishes. CEO/HNT nanoparticles were compressed into slices with a diameter of 10 mm and stabilized at the center of the petri dish lid, ensuring that the slice did not come into contact with the agar surface. To prevent the release of CEOs into the environment, the petri dishes were sealed hermetically. The dishes were then placed in a temperature-controlled incubator at 37 °C and incubated for 24 h.

#### 2.4.8. Assay of Strawberry Packaging

Fresh and undamaged strawberries were selected to evaluate the freshness-retaining effect of the composite films. Strawberries were wrapped using either PBAT or composite films. The packed strawberries were then placed into a constant-temperature and humidity chamber, with the conditions of 30 °C and 90% RH. Strawberries without any packaging served as the control group.

## 3. Results and Discussion

### 3.1. FT-IR Spectroscopy

**Figure 1:**
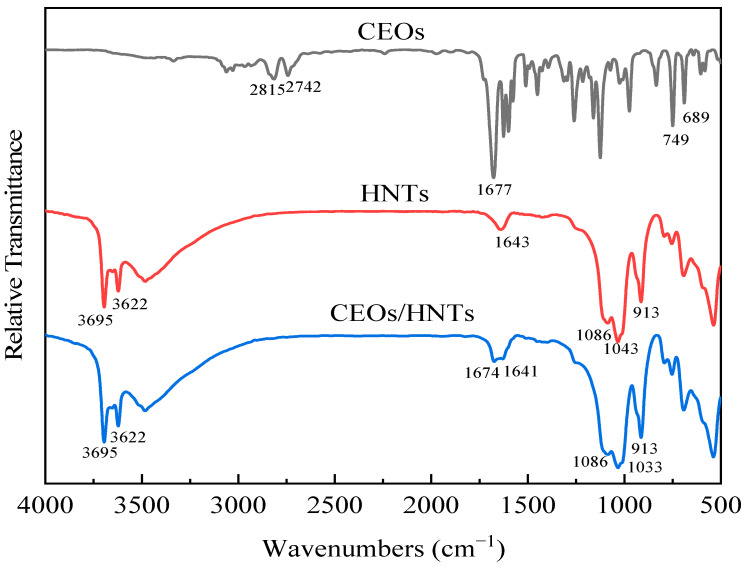
FT-IR spectra of CEOs, HNTs, CEOs/HNTs.

As shown in Figure 1, the stretching vibration peaks of the aldehyde group C-H inCEOs appear at 2815 cm^−1^ and 2742 cm^−1^; while the stretching vibration peak of C=O in the aldehyde group appeared at 1677 cm^−1^. The out-of-plane bending vibration peaks of C-H in the benzene ring were observed at 749 cm^−1^ and 689 cm^−1^ in CEOs [21]. This indicated that the cinnamyl aldehyde group is essentially present in CEOs. In the halloysite, the peaks at 3695 cm^−1^ and 3622 cm^−1^ correspond to the stretching vibration of internal hydroxyl groups on the shared surface and the stretching vibration of hydroxyl groups on the unshared surface of the layered structure, which consisted of S-O tetrahedra and Al-O octahedra, respectively. The adjacent absorption peaks suggested the presence of hydrogen bonds between the hydroxyl groups and the interlayer water. Additionally, bending vibration peaks of water molecules in halloysite were observed at 1643 cm^−1^, and stretching vibration peaks of Si–O appear at 1086 cm^−1^ and 1033 cm^−1^. A bending vibration peak of –OH was found at 913 cm^−1^ [22,23]. For CEOs/HNTs, the original halloysite characteristics remain, with a new peak appearing at 1674 cm^−1^, corresponding to the C=O stretching vibration peak of the aldehyde group in CEOs. This suggested that cinnamon essential oils were successfully loaded into the halloysite nanotubes.

### 3.2. Essential Oil-Loading and Retention Analysis

**Figure 2:**
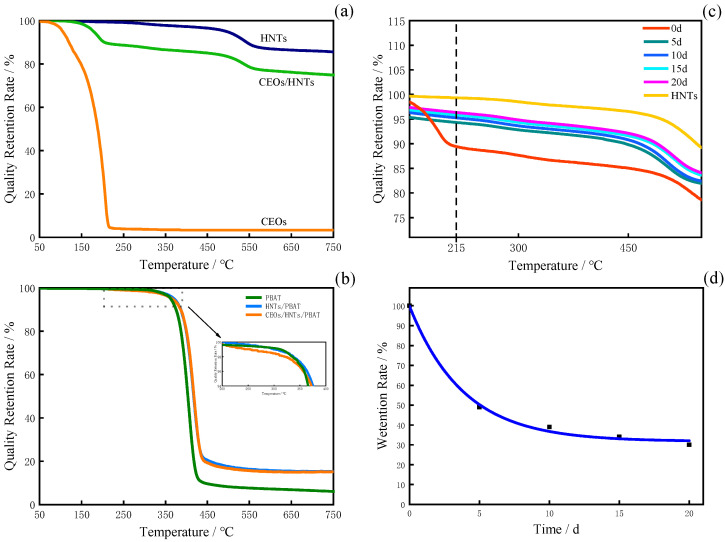
TG curves of (**a**) pure CEOs, HNTs, CEOs/HNTs, (**b**) pure PBAT films, HNTs/PBAT composite films, CEOs/HNTs/PBAT composite films, (**c**) TG of CEOs/HNTs at different times and (**d**) Retention rates of CEOs/HNTs at different times.

In order to accurately evaluate how much CEOs were loaded or retained in HNTs during the different storage periods, thermogravimetric analysis was employed here (see Figure 2a). The mass loss of HNTs primarily occurred between 450 °C and 600 °C, with a mass loss of approximately 8.5%, which was attributed to the dehydration of hydroxyl groups in HNTs [24]. The TG curve of CEOs began to decrease at 50 °C, which was probably due to the loss of volatile cinnamon essential oils. Subsequently, the quality of cinnamon essential oils remained relatively stable with increasing temperature until a pronounced inflection point at 215 °C, indicating the complete decomposition of cinnamon essential oils [25]. The TG curve of CEOs/HNTs exhibited two distinct weight loss intervals, at 100–220 °C and 450–550 °C, respectively. The initial weight loss was mainly attributed to the release of cinnamon essential oils from the halloysite nanotubes, with an estimated mass loss of approximately 11%, suggesting that the loading efficiency of cinnamon essential oils was around 11%. In the later stage, the mass loss was primarily due to the dehydration of hydroxyl groups in HNTs, leading to a decrease in mass.

The essential oils in HNTs will undergo some volatility due to the heat during plastic processing. It is necessary to determine the accurate amount of essential oil in the final film. Figure 2b shows the TGA curves of pure PBAT, HNTs/PBAT, and CEOs/HNTs/PBAT. The mass loss for both the HNTs/PBAT flexible film and the composite films primarily occurred between 350 °C and 450 °C, which was attributed to the degradation of PBAT resin [26]; this can also be seen in PBAT’s TGA curve. Within the range of 200–400 °C, the composite films showed a distinct downward trend, with a mass loss of about 0.4% compared to the HNTs/PBAT flexible film without essential oil. In the original formula, the content of cinnamon essential oils in the flexible film (CEO/HNT nanoparticles:PBAT = 1:9) theoretically should be around 1%. This meant that 40% of the absolute essential oil in CEO/HNT nanoparticles would be lost during the heat treatment process. The thermal stability of the composite films was enhanced after the incorporation of CEO/HNT nanoparticles. For further details, please refer to Section S2.3 in the supplementary materials [27,28,29,30].

According to the TG curve of CEOs in Figure 2b, it can be seen that when the temperature reaches 215 °C, the mass of CEOs basically no longer changes. Therefore, by comparing the mass loss of antibacterial nanoparticles at 215 °C on different placement days, the retention rate of CEOs in antibacterial nanoparticles on different placement days can be obtained. The mass fraction of CEOs in the initially prepared antibacterial nanoparticles is approximately 11% of the total mass. As the storage time increased, the cinnamon essential oils in the halloysite nanotubes were gradually released. The mass loss of CEO/HNT nanoparticles at 215 °C is shown in Figure 2c. The results indicated that the mass loss was 10.56%, 5.6%, 4.7%, 4.1%, and 3.6% on day 0, day 5, day 10, day 15, and day 20, respectively. Therefore, the retention rates of cinnamon essential oils encapsulated in CEO/HNT nanoparticles (the original CEO content was about 11%) were calculated to be 96%, 51%, 43%, 37%, and 33%, respectively. There are significant differences in the TGA curves of CEOs between day 0 and day 5, especially below 215 °C. This phenomenon can be attributed to the unique tubular structure of HNTs, which possess an extremely high specific surface area. After modification, the essential oils are not only encapsulated within the nanotube lumens but also adsorbed onto the extensive outer surfaces of the HNTs. During the heating process, in the samples on day 0, both the surface-adsorbed and lumen-encapsulated essential oils are decomposed. However, after day 5, the surface-adsorbed essential oils on the HNTs have completely volatilized, leaving only the lumen-encapsulated essential oils to be decomposed upon further heating. From Figure 2d, it can be seen that CEOs in HNT nanoparticles were rapidly released during the first 5 days, which was attributed to the evaporation of CEOs molecules from the surface and inside of HNTs.

We employed ultraviolet-visible (UV-Vis) spectrophotometry to indirectly determine the concentration variation of CEOs in ethanol solution, thereby enabling precise monitoring of the CEO release. CEOs exhibited a maximum absorbance at 284 nm and the fitted equation for the standard curve was y = 0.1672x − 0.0014, with an R^2^ value of 0.9996 (please refer to Figure S1a,b of the Supplementary Materials for the experimental data curve in this part). The release of CEOs was mainly divided into two stages, namely, the rapid release stage (0~7 d) and the sustained release stage (7~20 d). During the rapid release stage (0~7 d), the release of CEOs in the nanoparticles increased with the increase in time reached, and notably, the release rate of nanoparticles reached >50% at the 7th day. After the rapid release stage, the release of CEOs in the nanoparticles became slow and sustainable, and the release rate of nanoparticles reached 62.5%, 64.4% and 66.2% on the 10th, 15th and 20th days, with a daily release amount of 0.4%. In the stage of rapid release, the concentration of CEOs in the system could be increased to a relatively high level in a short time, which was beneficial for the rapid action of the antibacterial and fresh-keeping potency of the nanoparticles. After entering the slow and sustained release stage, the CEOs could be released at a daily rate of 0.4%, which endowed the system with a long-term release ability (the data curve of this part of the experiment is shown in Figure S2 in the Supplementary Materials). This pattern of rapid release in the initial stages followed by sustained release in the later stages was beneficial for the long-term antibacterial effectiveness of the antibacterial nanoparticles.

### 3.3. FE-SEM Analysis of Nanoparticle

**Figure 3:**
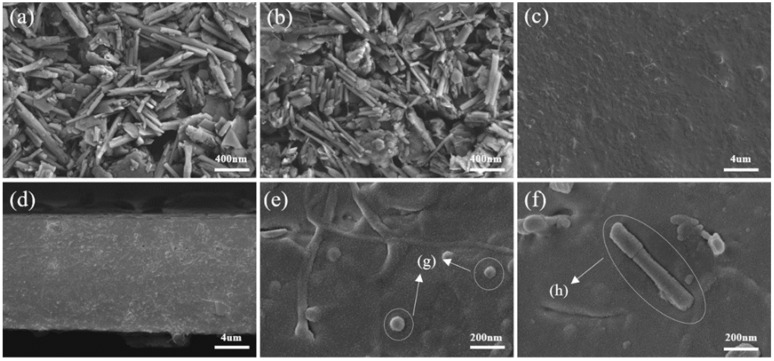
FE-SEM images of (**a**) HNTs before modification, (**b**) HNTs after modification, (**c**) the surface, and (**d**–**f**) cross-section of composite film.

Figure 3a illustrates the morphology of CEO/HNT nanoparticles. It was clearly observed that the halloysites were hollow tubular structures, with lengths ranging from 500 to 1500 nm and a nanotube diameter of approximately 50 to 100 nm. Notably, the tubular structures with open ends provided an opportunity for the successful loading of CEOs.

The morphology of the modified CEO/HNT nanoparticles (Figure 3b) exhibited a similar structure and size to that of the unmodified CEO/HNT nanoparticles. The adhesion of the modifier to the surface of the nanoparticles enhanced their compatibility with PBAT resin, facilitating the good dispersion of the CEO/HNT nanoparticles. The surface of the composite films appeared relatively smooth, with a few protruding white spots (Figure 3c), which were believed to be aggregated halloysite nanoclay particles [31]. The micro-morphology revealed that the modified CEO/HNT nanoparticles were uniformly distributed in the PBAT matrix resin. The cross-sectional morphology of the composite films (Figure 3d) also verified some micropores, likely resulting from the volatilization of cinnamon essential oils during the preparation process. Figure 3e,f shows the further enlarged cross-sectional morphologies of the CEOs/HNTs/PBAT films. In the figures, (g) represents the port of HNTs, and (h) represents the tubular body. This proves that the structure of HNTs remains intact in the films.

### 3.4. The Property Analysis of Films

**Light transmittance performance:** Table 1 shows the light transmittance of different films. Compared with the PBAT film, the light transmittance of the composite film decreased slightly. This phenomenon can be primarily attributed to the addition of HNTs into the PBAT resin, which reduced the transparency of the film as light was blocked by the halloysite in the film. Generally, food products should be stored in a dark environment; therefore, lower light transmittance in food packaging films can contribute to a longer shelf life of food [32].

**Water-permeability analysis of films:** As demonstrated in Table 1, compared with the control film, the water vapor permeability of the film slightly decreased after the addition of CEO/HNT nanoparticles. This is because the CEO/HNT nanoparticles in the film reduce the infiltration capacity of water molecules. The primary function of food packaging films was to regulate the transfer of water between the food and its surrounding environment. For certain types of food, packaging films with low water vapor permeability were desirable, as they can prevent the rapid growth of microorganisms due to high humidity within the packaging system, thereby extending the shelf life of the food.

**Oxygen-permeability analysis of films:** Many foods contain ingredients that are easily oxidized and deteriorated, making it essential to use packaging materials with oxygen barriers to extend their shelf life. The antibacterial composite film showed a 67% reduction in oxygen permeability compared to the PBAT film (Table 1). This is primarily due to the addition of CEOs/HNTs nanoparticles to the PBAT matrix resin. The nanoparticles caused the permeate to follow a longer pathway through the tortuous structure of the nanoclay. This reduction in oxygen permeability demonstrated the superior ability of the clay orientation to prevent oxygen from entering the packaging material, ultimately helping to extend the shelf life of food [33,34].

**Figure 4:**
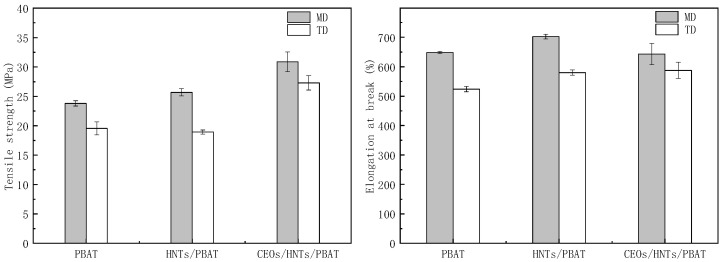
Tensile strength and elongation at break of different films; machine direction tensile strength (MD); transverse direction tensile strength (TD).

**Mechanical property of films:** As illustrated in Figure 4, the prepared composite film had excellent mechanical properties with tensile strength of above 18 Mpa. It was obvious that the machine direction tensile strength (MD) was higher than the transverse direction (TD) tensile strength, because during the film-blowing process, the low blowing ratio causes the polymer network to have a certain degree of orientation in the mechanical direction. The addition of HNTs to PBAT films enhanced the tensile strength. This was because the HNTs can effective transfer stress from the matrix PBAT resin to the nanofillers. The CEOs/HNTs/PBAT film presented the best tensile strength. A possible reason is that the absolute content of halloysite in the film was the lowest, which enabled optimal HNTs dispersion in the PBAT matrix resin. Notably, the elongation at break of CEOs/HNTs/PBAT film dropped to about 600% due to the plasticizing effect of the CEO/HNT nanoparticles in the PBAT matrix. However, this also benefited its practical use value.

### 3.5. Analysis of Antibacterial Activity of Composite Films

**Figure 5:**
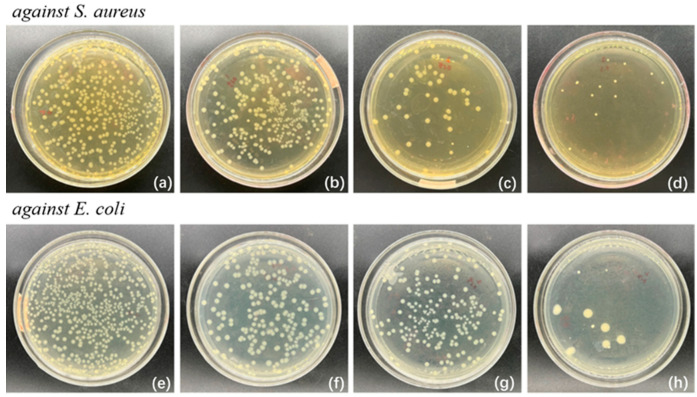
Antibacterial effects of the oil-loading nanoparticle composite film ((**a**–**d**) is against *S. aureus*, and (**e**–**h**) is against *E. coli.* The additive amounts of film is 0 g, 0.1 g, 0.15 g, 0.2 g from left to right).

Figure 5 illustrates the antibacterial effects of the composite films against *Staphylococcus aureus* (a~d) and *Escherichia coli* (e~h). It was obvious that the antibacterial effects became more pronounced as the amount of film increased. In particular, the bacterial colonies in the agar medium gradually diminished when the mass of the film increased to 0.2 g, which confirmed that the composite films exhibited strong antibacterial activity against *S. aureus* and *E. coli*. Specifically, the antibacterial rates against *S. aureus* reached 40%, 83%, and 93%, and for *E. coli*, the antibacterial rates rose to 50%, 67%, and 97%, when the mass of the composite films was 0.1 g, 0.15 g, and 0.2 g, respectively.

### 3.6. Analysis of the Non-Contact Bacteriostatic Zone of Antibacterial Particles

**Figure 6:**
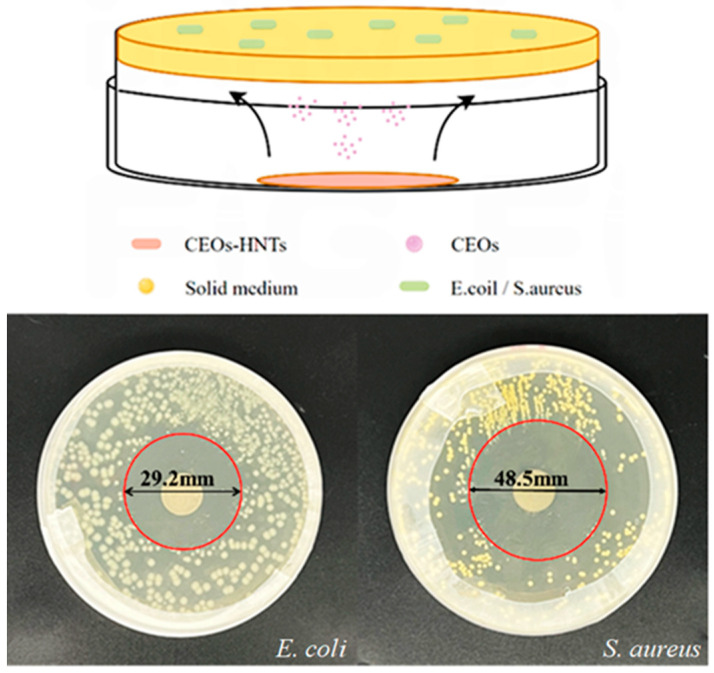
Schematic description of non-contact antimicrobial activity of the CEO/HNT nanoparticles.

CEOs primarily exert their antibacterial effect through molecular diffusion. The petri dish was incubated for 24 h, and the resulting effect is shown in Figure 6. In Figure 6, the two cultures are *E. coli* and *S. aureus*, respectively. It can be observed that, under the condition where the antibacterial particles do not directly contact the agar, there is a clear inhibition zone in both petri dishes. The size of the inhibition zone was 29.2 mm and 48.5 mm, respectively. This demonstrates that CEOs can exert an antibacterial effect through molecular diffusion, even in the absence of direct contact with the objects.

### 3.7. Freshness Preservation Applications of the CEOs/HNTs/PBAT Biodegradable Films

**Figure 7:**
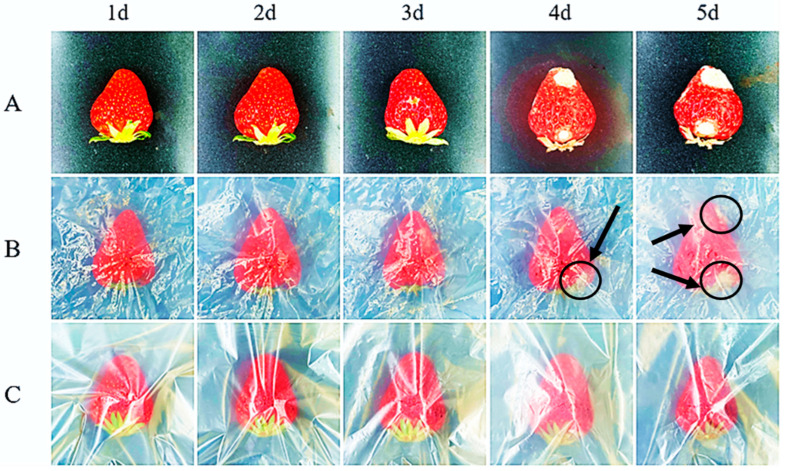
Changes in the appearance of strawberries in different packaging. ((**A**). unwrapped; (**B**). wrapped in the PBAT film; (**C**). wrapped in the CEOs/HNTs/PBAT biodegradable films).

In Figure 7, it was evident that the unwrapped strawberries, strawberries packaged with PBAT, and strawberries packaged with the composite films all maintained their initial appearance during the first two days. This was supported by the fact that the strawberries had a compact skin and bright surface color. Mildew spots began to appear on the surface of unwrapped strawberries on the third day. On the fifth day, nearly half of the surface of the strawberries had rotted, with significant plaque formation. On the fourth day, the strawberries packaged in PBAT film began to show mildew spots, which appeared later than in the unwrapped strawberries, and the overall deterioration was slightly less severe than that of the unwrapped ones. Strawberries packed with the composite films did not show any signs of mold growth. On the fifth day, the strawberries still maintained a good level of freshness, with firm skin, indicating that the CEO/HNT nanoparticles played an effective role in preserving the strawberries. This confirms that the composite films loaded with cinnamon essential oils has great potential and value for food preservation.

## 4. Conclusions

In this study, CEO/HNT nanoparticles were blended with PBAT matrix to produce degradable films with antibacterial properties. Cinnamon essential oils were successfully loaded into halloysite nanotubes with 11% loading efficiency. The CEO/HNT nanoparticles exhibited long-term sustained release characteristics. Composite films not only possess excellent mechanical strength but also present significant antibacterial ability. Additionally, composite film demonstrates a promising preservation effect in strawberry storage applications. This degradable film has potential for use in food packaging systems to enhance shelf life.

## Figures and Tables

**Table 1:** Light transmittance, water permeability, and oxygen permeability in different films.

Sample	LT (%)	WP (g/(m^2^·24 h))	OP (cm^3^/(m^2^·24 h·0.1 MPa))
PBAT	76 ± 1	186 ± 2	4123 ± 207
CEOs/HNTs/PBAT	71 ± 3	180 ± 1	1368 ± 101

Note: Values are expressed as mean ± standard deviation (n = 3) (LT: light transmittance; WP: water permeability; OP: oxygen permeability).

## Data Availability

The original contributions presented in this study are included in the article/supplementary material. Further inquiries can be directed to the corresponding author.

## Supplementary Materials

The following supporting information can be downloaded at: https://www.mdpi.com/article/10.3390/polym17091144/s1, Figure S1: (a) Wavelength scanning curve and (b) standard curve of CEOs; Figure S2: Change of CEOs concentration over time; Figure S3: DTG curves of HNTs, CEOs, HNTs/CEOs (a) and HNTs/CEOs nanoparticles at different time periods (b); Figure S4: TG curves (a) and DTG curves (b) of pure PBAT, HNTs/PBAT, CEOs/HNTs/PBAT. Table S1: The mechanical properties of PBAT films on the mechanical direction (MD);Table S2: The mechanical properties of PBAT films on the transverse direction (TD); Table S3: The mechanical properties of HNTs/PBAT films on the mechanical direction (MD); Table S4: The mechanical properties of HNTs/PBAT films on the transverse direction (TD); Table S5: The mechanical properties of CEOs/HNTs/PBAT films on the mechanical direction (MD); Table S6: The mechanical properties of CEOs/HNTs/PBAT films on the transverse direction (TD).
