# Supplementary material for: Cinnamon Essential Oil-Loaded Halloysite Nanotubes Applied in Degradable Film: Characterization and Non-Contact Antimicrobial Activity
Source: Polymers (Basel). 2025 Apr 23;17(9):1144. doi: 10.3390/polym17091144 (PMC12074084; doi:10.3390/polym17091144)
Supplement: Supplementary file 1 [file polymers-17-01144-s001.zip › polymers-3491392-supplementary.pdf]

Supplementary Materials

The supporting material mainly includes slow release rate study, data supplement of mechanical properties experiment of the film, differential thermogravimetric (DTG) analysis. The experiment of slow release rate verifies that the system has sustained and stable long-term release potential. The experimental results of mechanical properties show that the film exhibits excellent mechanical properties in both transverse and longitudinal tensile directions. Through DTG analysis, we determined that the addition of CEOs enhanced the thermal stability of the film, providing a solid experimental basis and broad development prospects for its wide application in various fields.

1. Characterization

1.1 Determination of sustained release rate of nanoparticles

The maximum light absorption wavelength of cinnamon essential oils (CEOs) within the range of 200–900 nm was determined using a UV-Vis spectrophotometer. A series of CEOs solutions with concentrations of 1, 2, 3, 4, and 5 μg/mL were prepared using anhydrous ethanol. Under these conditions, the absorbance values of the CEOs solutions at different concentration gradients were measured with the spectrophotometer, and a standard curve was constructed. To evaluate the release behavior of CEOs from the CEOs/HNTs nanoparticles, 0.1 g of the nanoparticles was weighed and placed into seven centrifuge tubes. Each tube was filled with 10 mL of anhydrous ethanol and left to stand at room temperature. At specified intervals—on the 1st, 2nd, 4th, 7th, 10th, 15th, and 20th days—the supernatant from each tube was collected, centrifuged, diluted to an appropriate concentration, and its absorbance was measured. Using the CEOs standard curve, the CEOs concentration in the supernatant at each time point was calculated.

1.2 Thermal Stability Analysis of Films

The loading efficiency of CEOs in PBAT films was analyzed and determined using a synchronous thermal analyzer (TGA-5500, TA Instruments, USA). Approximately 5 milligrams of each sample were weighed and placed in an alumina crucible. The analysis was conducted under a nitrogen atmosphere with a nitrogen flow rate of 40 mL/min. The temperature range was from 40°C to 800°C, and the heating rate was 20°C/min.

2. Results and discussion

2.1 The slow-release property of halloysite nanoparticles

This study employed ultraviolet-visible (UV-Vis) spectrophotometry to indirectly determine the concentration variation of CEOs in ethanol solution. During the experiment, CEOs were continuously released from the HNTs carrier and dissolved in the ethanol medium, resulting in a dynamic increase in CEOs concentration over time. Due to the technical challenges associated with direct quantitative analysis of CEOs concentration, the absorbance at a characteristic wavelength was measured to indirectly characterize the CEOs concentration in the solution. This method is based on the Lambert-Beer's law, which states that the absorbance of a solution at a specific wavelength is linearly correlated with the solute concentration. Thus, a quantitative relationship between absorbance and CEOs concentration was established, enabling precise monitoring of the CEOs release from HNTs.

In Figure S1(a), CEOs exhibited a maximum absorbance at 284 nm. The standard curve, shown in Figure S1(b), was constructed by plotting the concentrations of CEOs on the x-axis and the absorbance values on the y-axis. The fitted equation for the standard curve was $y=0.1672x-0.0014$, with an R^2 value of 0.9996.

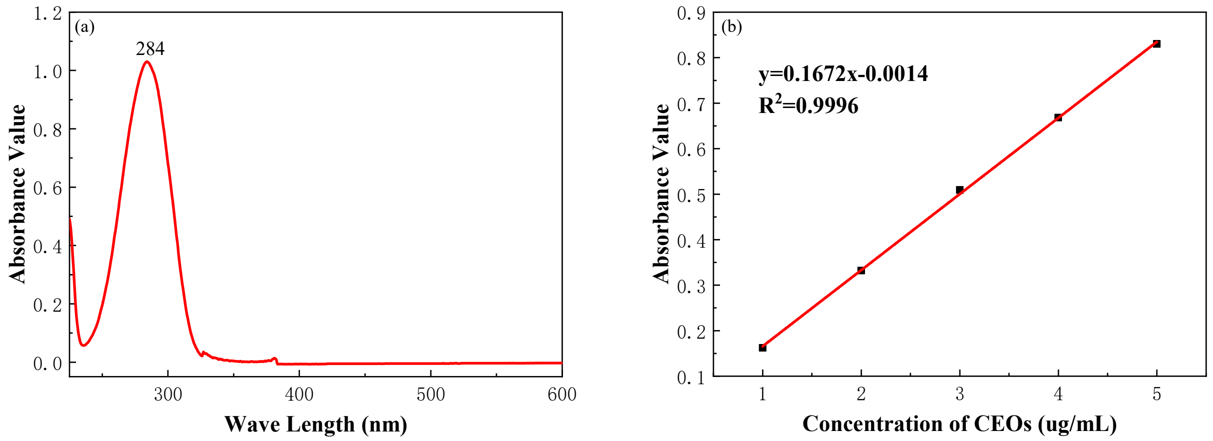

Figure S1 (a)Wavelength scanning curve and (b)standard curve of CEOs

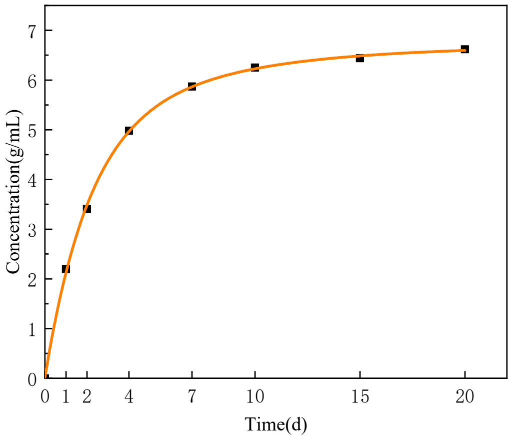

Figure S2 Change of CEOs concentration over time

2.2 Mechanical property analysis of films

Table S1~6 shows a supplementary table of mechanical properties

Table S1 The mechanical properties of PBAT films on the mechanical direction (MD)

PBAT MD	tensile strength/Mpa	elongation at break/%
1	**23.45**	**650.49**
2	**24.29**	**649.75**
3	**23.67**	**644.30**
Arithmetic mean	23.803	648.180
standard deviation	0.356	2.760
standard error	0.126	0.976

Table S2 The mechanical properties of PBAT films on the transverse direction (TD)

PBAT TD	tensile strength/Mpa	elongation at break/%
1	**20.38**	**531.50**
2	**18.30**	**513.70**
3	**19.97**	**526.43**
Arithmetic mean	19.550	523.877
standard deviation	0.900	7.488
standard error	0.318	2.647

Table S3 The mechanical properties of HNTs/PBAT films on the mechanical direction (MD)

HNTs/PBAT MD	tensile strength/Mpa	elongation at break/%
1	**26.27**	**710.71**
2	**25.02**	**694.48**
3	**25.78**	**702.59**
Arithmetic mean	25.690	702.593
standard deviation	0.514	6.626
standard error	0.182	2.343

Table S4 The mechanical properties of HNTs/PBAT films on the transverse direction (TD)

HNTs/PBAT TD	tensile strength/Mpa	elongation at break/%
1	**18.63**	**583.44**
2	**18.86**	**569.66**
3	**19.32**	**586.70**
Arithmetic mean	18.937	579.933
standard deviation	0.287	7.385
standard error	0.101	2.611

Table S5 The mechanical properties of CEOs/HNTs/PBAT films on the mechanical direction (MD)

CEOs/HNTs/PBAT MD	tensile strength/Mpa	elongation at break/%
1	**29.53**	**627.90**
2	**30.34**	**617.25**
3	**32.76**	**684.36**
Arithmetic mean	30.877	643.170
standard deviation	1.372	29.448
standard error	0.485	10.412

Table S6 The mechanical properties of CEOs/HNTs/PBAT films on the transverse direction (TD)

CEOs/HNTs/PBAT TD	tensile strength/Mpa	elongation at break/%
1	**26.55**	**538.96**
2	**26.62**	**544.89**
3	**28.70**	**589.56**
Arithmetic mean	27.290	557.803
standard deviation	0.997	22.585
standard error	0.353	7.985

2.3 Differential thermogravimetric (DTG) analysis of antibacterial nanoparticles and films

Differential Thermogravimetric (DTG) analysis is a powerful technique used to study the thermal decomposition behavior of materials. In the context of antibacterial films, DTG analysis can provide valuable insights into the composition, thermal stability, and the release mechanisms of antibacterial agents incorporated within the film matrix.

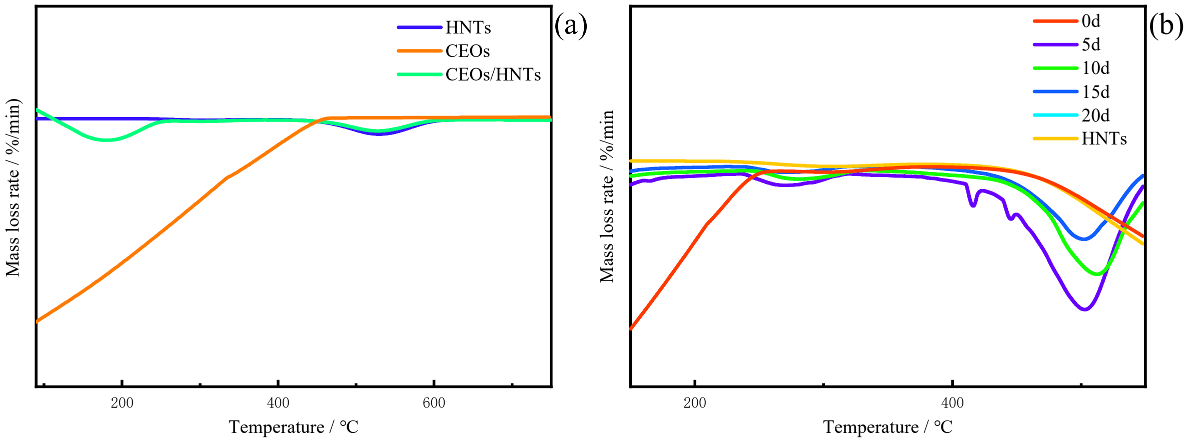

Figure S3. DTG curves of HNTs, CEOs, HNTs/CEOs (a) and HNTs/CEOs nanoparticles at different time periods (b)

As shown in Figure 3(a), both HNTs and CEOs have a single decomposition phase. In contrast, CEOs /HNTs nanoparticles exhibit two decomposition stages, the thermal decomposition of CEOs and HNTs. Figure 3(b) shows that the composite film undergoes multiple thermal decomposition stages. It is worth noting that with the extension of storage time, the peak strength gradually decreases, which can be attributed to the gradual release of CEOs from the film and the reduction of ceos content in the film, resulting in a flattening of the DTG curve.

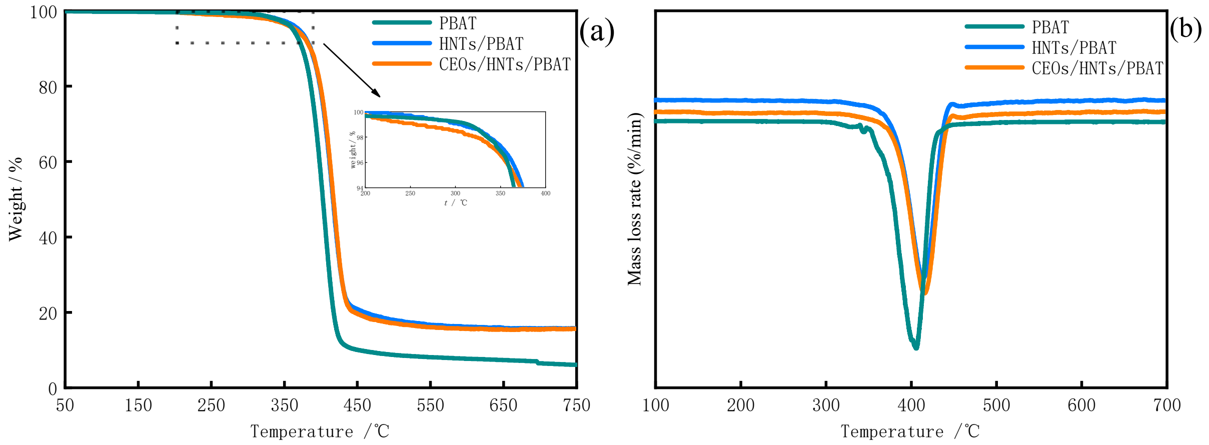

Figure S4. TG curves (a) and DTG curves (b) of pure PBAT, HNTs/PBAT,CEOs/HNTs/PBAT

A first-order derivative of Fig. 4(a) was performed to obtain Fig. 4(b), aiming to explore the influence of the addition of CEOs on the thermal decomposition rate of the composite film. As shown in Figure 4(b), the highest thermal decomposition rate temperature (Tp) of pure PBAT film is about 407°C, the highest thermal decomposition rate temperature of HNTs/PBAT composite film is about 416°C, and the highest thermal decomposition rate temperature of ceo /HNTs/PBAT composite film is about 418°C. The addition of a small amount of CEOs/HNTs nanoparticles is conducive to increasing the maximum thermal decomposition rate temperature of the composite film, thereby enhancing its thermal stability. This is because in composite films, CEOs exist in the form of nanoparticles, and carrier materials (HNTs) can protect CEOs and make them more stable at high temperatures[1-4].

References

[1] Hu J, Zhang Y, Xiao Z, et al. Preparation and properties of cinnamon-thyme-ginger composite essential oil nanocapsules[J]. Industrial crops and products, 2018, 122: 85-92.

[2] Yang N, Ashton J, Kasapis S. The influence of chitosan on the structural properties of whey

protein and wheat starch composite systems[J]. Food Chemistry, 2015, 179: 60-67.

[3] Zhou D, Pan Y, Ye J, et al. Preparation of walnut oil microcapsules employing soybean protein isolate and maltodextrin with enhanced oxidation stability of walnut oil[J]. LWT-Food Science and Technology, 2017, 83: 292-297.

[4] Yang K, Liu A, Hu A, et al. Preparation and characterization of cinnamon essential oil nanocapsules and comparison of volatile components and antibacterial ability of cinnamon essential oil before and after encapsulation[J]. Food control, 2021, 123: 107783.
